# Over-expression of Thrombospondin 4 correlates with loss of miR-142 and contributes to migration and vascular invasion of advanced hepatocellular carcinoma

**DOI:** 10.18632/oncotarget.15054

**Published:** 2017-02-03

**Authors:** Fang Su, Jun Zhao, Shukui Qin, Rui Wang, Yumei Li, Qiang Wang, Yi Tan, Hao Jin, Fangquan Zhu, Yurong Ou, Zenong Cheng, Wen Su, Fuyou Zhao, Yan Yang, Zhengguang Zhou, Jiyue Zheng, Yawei Li, Zhongwen Li, Qiong Wu

**Affiliations:** ^1^ Department of Medical Oncology, First Affiliated Hospital of Bengbu Medical College, Bengbu 233004, Anhui, China; ^2^ Department of General Surgery, Yijishan Hospital of Wannan Medical College, Wuhu 241000, Anhui, China; ^3^ Department of Medical Oncology, PLA Cancer Center, Nanjing Bayi Hospital, Nanjing 210002, Jiangsu, China; ^4^ Department of Information Center, Bengbu Medical College, Bengbu 233030, Anhui, China; ^5^ Department of Hepatobiliary Surgery, First Affiliated Hospital of Bengbu Medical College, Bengbu 233004, Anhui, China; ^6^ Departments of Pathology, First Affiliated Hospital of Bengbu Medical College, Bengbu 233004, Anhui, China; ^7^ Department of Biologic Science, Bengbu Medical College, Bengbu 233030, Anhui, China

**Keywords:** THBS4, miR-142, hepatocellular carcinoma, migration, vascular invasion

## Abstract

Hepatocellular carcinoma (HCC) is a common malignancy found worldwide and is associated with a high incidence of metastasis and vascular invasion. Elucidating the molecular mechanisms that underlie HCC tumorigenesis and progression is necessary for the development of novel therapeutics. By analyzing the Cancer Genome Atlas Network (TCGA) dataset, we identified Thrombospondin 4 (THBS4) is significantly overexpressed in HCC samples and is correlated with prognosis. Overexpression of THBS4 was also highly correlated with vascular invasion of advanced HCC. While THBS4 is often overexpressed in HCC it has also been shown to inhibit tumor growth by mediating cell-to-cell and cell-to-matrix interactions. Here, we identified that knockdown of THBS4 inhibits migration and invasion of HCC cells and inhibits HCC induced angiogenesis. MiRNAs are crucial regulators of multiple cellular processes, and aberrant expression of miRNAs has been observed to effect cancer development and progression. We further found that miR-142 is an upstream regulator of THBS4 in HCC cells. Moreover, miR-142 was significantly down-regulated in HCC tissue samples and correlated with overexpression of THBS4. Overexpression of miR-142 inhibited invasion and angiogenesis of HCC cells and re-expression of THBS4 overcame these effects of miR-142 expression. Stable over-expression of miR-142 significantly inhibited tumour growth in a xenograft tumour model through inhibiting THBS4 expression and tumor angiogenesis. In conclusion, our findings indicate that loss of miR-142 results in the over-expression of THBS4, which enhances HCC migration and vascular invasion. Thus, targeting THBS4 or miR-142 may provide a promising therapeutic strategy for treatment of advanced HCC.

## INTRODUCTION

HCC ranks as the fifth most common cancer worldwide and the third most common cause of cancer mortality. It causes approximately 24,550 new deaths, accounting for 4% of all the cancer-related deaths in the United States during 2015 [1]. HCC is characterized by a high degree of heterogeneity and potential for invasion and migration into adjacent normal tissues [2–4]. The median overall survival of patients who are diagnosed with HCC is less than one year due to the absence of effective treatments [5]. Although a number of potential therapeutic targets have been investigated, such as receptor tyrosine kinases (RTKs) and anti-angiogenesis antibodies, the long-term outcome of HCC following these treatment strategies remains unclear [6–8]. Therefore, further understanding of the underlying molecular mechanism should provide further impetus for the development of novel and effective therapeutic strategies for HCC patients.

MicroRNAs (miRs) are small, non-coding RNAs which have the ability to regulate the expression of many genes at both the transcriptional and translational level [9]. Accumulating evidence suggested that miRs may act as oncogenes or tumor suppressor genes by regulating their targets, most of which were the key regulators in the process of cell proliferation, apoptosis, metastasis and angiogenesis [10]. Previous studies have identified that miR-142 plays a critical role in the development of a variety of cancers such as osteosarcoma and renal cancer [11, 12]. However, the expression and function of miR-142 in HCC still remains unclear.

Thrombospondin 4 (THBS4), an extracellular calcium-binding proteins, forms part of the extracellular matrix and plays an important role in cellular invasion, migration, adhesion and attachment [13, 14]. Recent studies have identified THBS4 as a regulatory of multiple cancers, such as prostate cancer, breast cancer and some types of gastric cancers [13–16]. However, it has not been detected in HCC and whether THBS4 contributes to migration and vascular invasion of advanced HCC remains unknown.

In this study, we characterized the expression of THBS4 and miR-142 in HCC cell lines and tumor samples. We identified that miR-142 functions to regulate the expression of THBS4, and loss of miR-142 promotes invasion and migration of HCC through upregulation of THBS4. Furthermore, we found that THBS4 expression was significantly correlated with the prognosis of HCC patients, indicating that both THBS4 and miR-142 may be promising targets for therapy and/or prognostic markers of HCC.

## RESULTS

### THBS4 is overexpressed in HCC and associated with vascular invasion and survival

**Figure 1:**
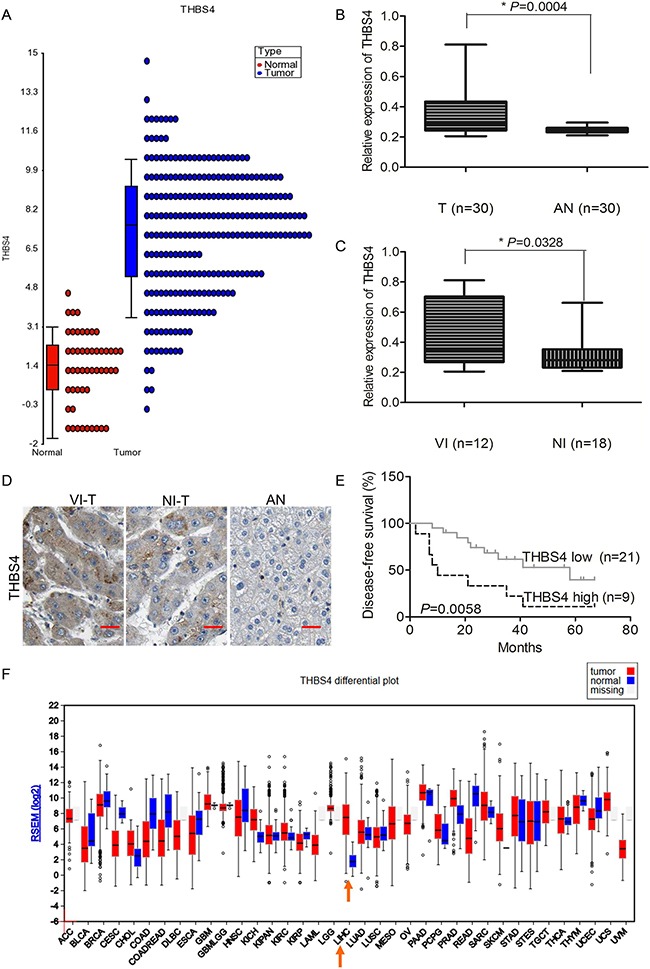
THBS4 is overexpressed in HCC and associated with vascular invasion and survival **A**. Relative expression level of *THBS4* in HCC tumor and non-tumor samples in TCGA dataset. **B**. Relative expression of *THBS4* in 30 pairs of HCC and adjacent normal tissue by RT-qPCR analysis (T: tumor, AN: adjacent normal). **C**. Relative expression of *THBS4* in 30 pairs of HCC samples with vascular invasion (VI) and non-vascular invasion (NI). **D**. Representative images of THBS4 in HCC tumors with vascular invasion (VI-T) and non-vascular invasion (NI-T) and adjacent normal tissues (AN). **E**. Association of *THBS4* expression with patient survival. **F**. Expression of *THBS4* in TCGA dataset.

By analyzing HCC tumor samples in the TCGA database, we found that THBS4 expression was significantly higher in HCC tumor samples compared with adjacent normal samples (Figure 1a). To validate the expression of THBS4 in clinical tumor samples, 30 pairs of HCC and adjacent normal samples were analyzed by qRT-PCR. Consistent with the TCGA dataset, THBS4 was higher in the HCC samples than adjacent normal samples (Figure 1b). Furthermore, we divided the 30 HCC tumor samples into two groups according to whether they had vascular invasion or not and observed that the tumors that showed vascular invasion had higher expression of THBS4 than those tumors which did not exhibit vascular invasion (Figure 1c). The expression of THBS4 in each group (tumors with vascular invasion, tumors without vascular invasion, and adjacent normal tissue) was also detected using IHC, and showed consistent results with those obtained from qRT-PCR analysis (Figure 1d).

To assess if expression of THBS4 was associated with overall patient survival, we separated the 30 HCC samples into high expression and low expression of THBS4 according to the results from qRT-PCR. The average expression of THBS4 across all samples was utilized as the cut off value. The results showed that the group that expressed higher THBS4 had shorter overall survival (Figure 1e). In addition, the TGCA database also showed that HCC had a higher expression of THBS4 (Figure 1f). (Expression of THBS1, THBS2, THBS3 and THBS5 in HCC of TCGA dataset was shown in Supplementary Figure 1.)

### Loss of THBS4 inhibits migration, invasion and angiogenesis of HCC cells

**Figure 2:**
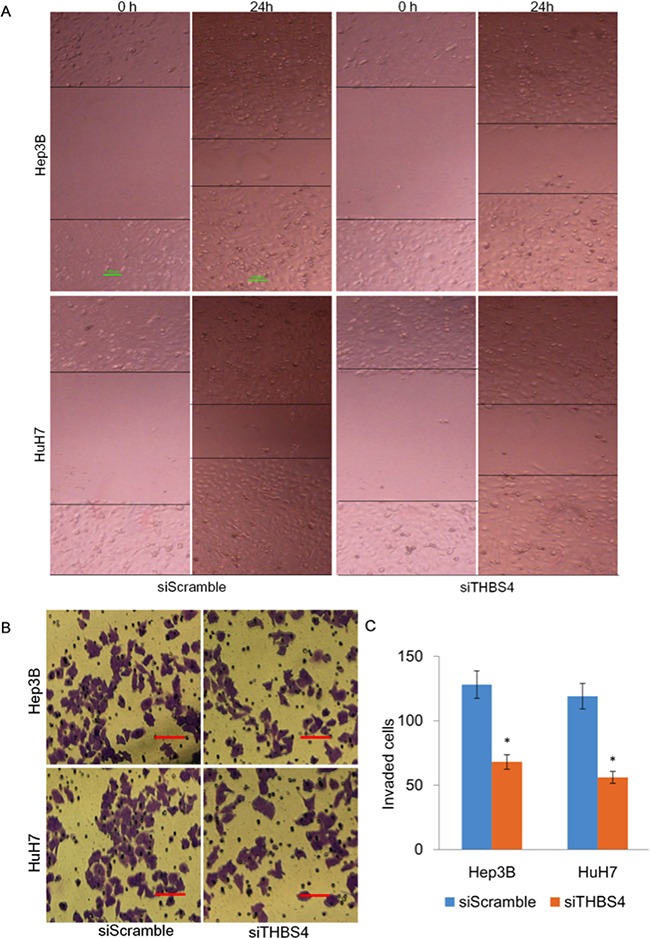
Knockdown of THBS4 inhibits migration and invasion of HCC cells **A**. Representative images of cell migration using wound healing assay. **B**. Representative images of transwell assays measuring *in vitro* Matrigel cell invasion, following depletion of THBS4 in HCC cells. **C**. Quantification of invasion assay. The invading cells were quantified by plotted as average plotting them as the average number of cells per field of view from 3 different independent experiments as described.

Given our initial observation that THBS4 expression correlated with tumor invasiveness, we investigated whether THBS4 regulated cellular migration and invasion *in vitro*. To assess the role of THBS4 in HCC cell migration, we depleted THBS4 in HuH7 and Hep3B cells using siTHBS4. Using wound healing assays to evaluate the cellular migration, we found that depletion of THBS4 significantly reduced cell migration in both HuH7 and Hep3B cells (Figure 2a). To assess the role of THBS4 in HCC cell invasion, we utilized transwell assays in HuH7 and Hep3B cells expressing siTHBS4. Compared with cells transfected with siScramble, depletion of THBS4 repressed the invasion capacity of HuH7 and Hep3B cells (Figure 2b–2c). These results indicate that THBS4 expression levels are critically important for cellular migration and invasion.

**Figure 3:**
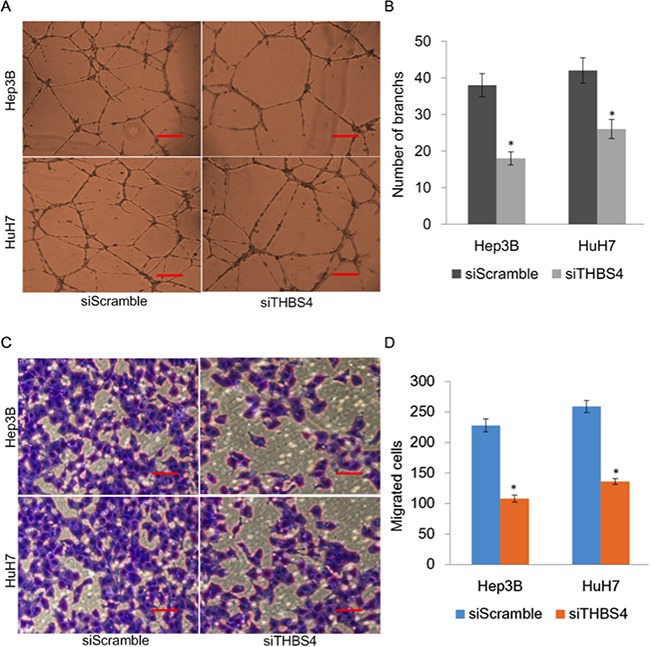
Knockdown of THBS4 inhibits HCC cells induced angiogenesis **A**. Representative tube formation by endothelial cells after incubation with conditioned media (CM) from HuH7 and Hep3B cells transfected with siScrmble or siTHBS4 using the tube formation assay. **B**. Quantification of the number of branches in each group. **C**. Representative images of endothelial cell migration after incubation with conditioned media (CM) from HuH7 and Hep3B cells transfected with siScramble or siTHBS4 using the endothelial recruitment assay. **D**. Quantification of the numbers of migrating endothelial cells in each group.

To further evaluate the effect of THBS4 expression and angiogenesis, we assessed tube formation of endothelial cells which were incubated with CM from HuH7 and Hep3B cells transfected with siScrmble or siTHBS4 (Figure 3a). We observed that endothelial cell tube formation in cells transfected with siTHBS4 was significantly less than cells transfected with control siScramble (Figure 3b). Endothelial cell migration was further determined using an endothelial recruitment assay which demonstrated that depletion of THBS4 inhibited the migration of endothelial cells (Figure 3c and 3d).

### miR-142 is a upstream regulator of THBS4 in HCC cells

**Figure 4:**
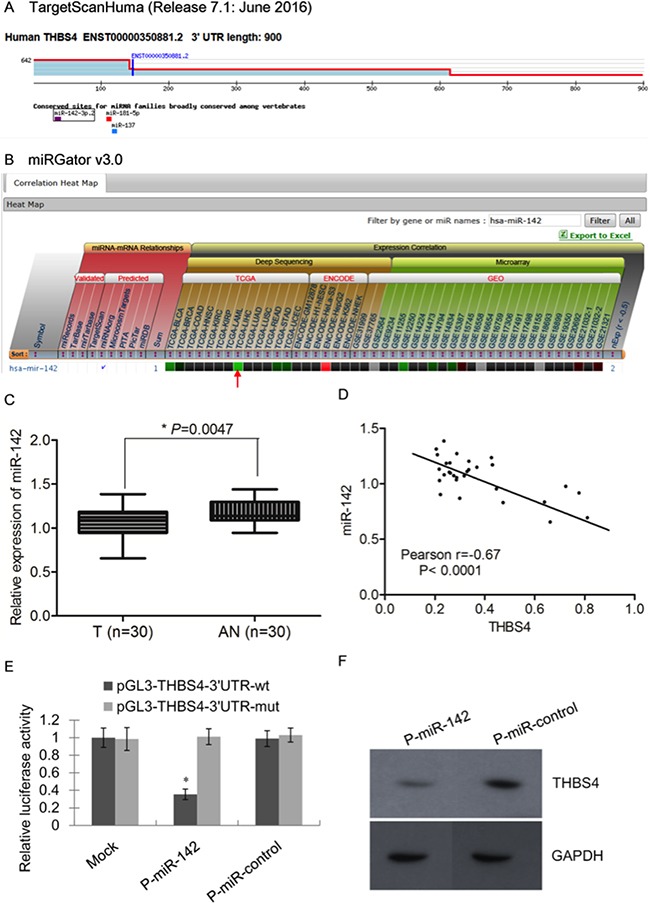
miR-142 is an upstream regulator of THBS4 in HCC cells **A**. Putative microRNAs targeting *THBS4* as predicted by TargetScan. **B**. The prediction and correlation heat map in different dataset by miRGator 3.0. **C**. Relative expression of miR-142 in 30 pairs of HCC and adjacent normal samples by RT-qPCR analysis (T: tumor, AN: adjacent normal). **D**. Inverse correlation of *THBS4* and miR-142 in a group of HCC samples based on the RT-qPCR analysis. **E**. HuH7 cells co-transfected with P-miR-142 with wild-type (wt) or mutant (mut) pGL3-THBS4 constructs followed by luciferase reporter assays. Data were normalized by the ratio of Firefly and Renilla luciferase activities measured at 48 h post-transfection. The bar graph shows the mean ±SD in three independent transfection experiments. *P<0.05. **F**. Western blotting analysis of THBS4 expression in P-miR-control and P-miR-142-transfected HuH7 cells.

MiRs have been shown to function during tumorigenesis by regulating expression of oncogenes and tumor suppressors to effect critical cellular processes including cell proliferation, apoptosis, metastasis and angiogenesis [10]. In order to investigate the possible regulation of THBS4 by miRs in HCC, we used the online bioinformatics database TargetScan to identify miRs that may target THBS4. We found that miR-142-3p.2, miR-181-5p and miR-137 possess seed sequences that could target *THBS4* mRNA (Figure 4a). Further analysis using miRGator 3.0 showed a negative correlation of THBS4 with miR-142 but not miR-181 and miR-137 (Figure 4b). Therefore, we evaluated the expression of miR-142 in the 30 HCC samples and their adjacent normal samples and found that the expression of miR-142 in HCC samples was significantly lower than that of adjacent normal samples (Figure 4c). Pearson correlation analysis demonstrated that the expression of THBS4 and miR-142 were significantly inversely correlated based on qRT-PCR analysis (Figure 4d). These data suggest that miR-142 may reduce THBS4 expression to suppress HCC tumorigenesis.

To further assess the ability of miR-142 to directly suppress *THBS4* expression, we utilized luciferase reporter assays. HuH7 cells expressing either the wild-type or miR-142 seed sequence mutant *THBS4* 3`UTR (pGL3-THBS4) linked to luciferase were co-transfected with p-miR-142. Results showed that pGL3-THBS4-3`UTR-wt HuH7 cells had a significantly lower expression of miR-142 after transfected with p-miR-142 compared with p-miR-control (Figure 4e). Furthermore, western blotting analysis of HCC tumor samples showed that expression of THBS4 was inversely correlated with miR-142 (Figure 4f).

### Forced expression of miR-142 attenuates the invasion and angiogenesis of HCC cells

**Figure 5:**
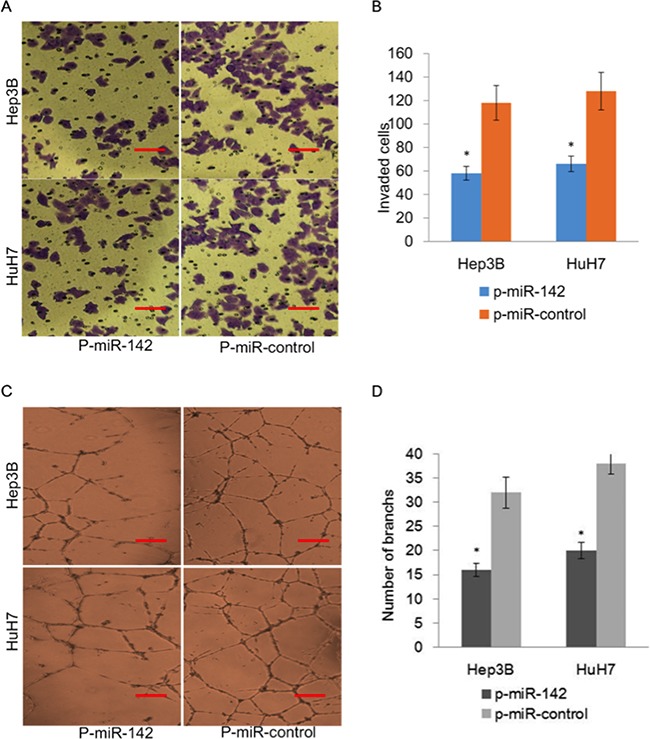
Overexpression of miR-142 inhibits invasion and angiogenesis of HCC cells **A**. Representative images of transwell *in vitro* Matrigel cell invasion following overexpression of miR-142 in HCC cells. **B**. Quantification of invading cells plotted as the average number of cells per field of view from 3 independent experiments as described. **C**. Representative tube formation by endothelial cells after incubation with conditioned media (CM) from HuH7 and Hep3B cells transfected with p-miR-control or p-miR-142 using the tube formation assay. **D**. Quantification of the number of branches in each group.

To further assess the role of miR-142 in angiogenesis, we ectopically expressed p-miR-142 in HuH7 and Hep3B cells and performed transwell and endothelial tube formation assays. Our results indicated that ectopic expression of miR-142 inhibited the invasiveness of HuH7 and Hep3B cells as measured utilizing a transwell assay (Figure 5a and 5b). Furthermore, ectopic expression of miR-142 suppressed endothelial tube formation after incubation with conditioned media (CM) and suppressed the migration of endothelial cells in comparison with vector control (Figure 5c and 5d).

### miR-142 inhibits tumor formation of HCC cells through targeting THBS4

**Figure 6:**
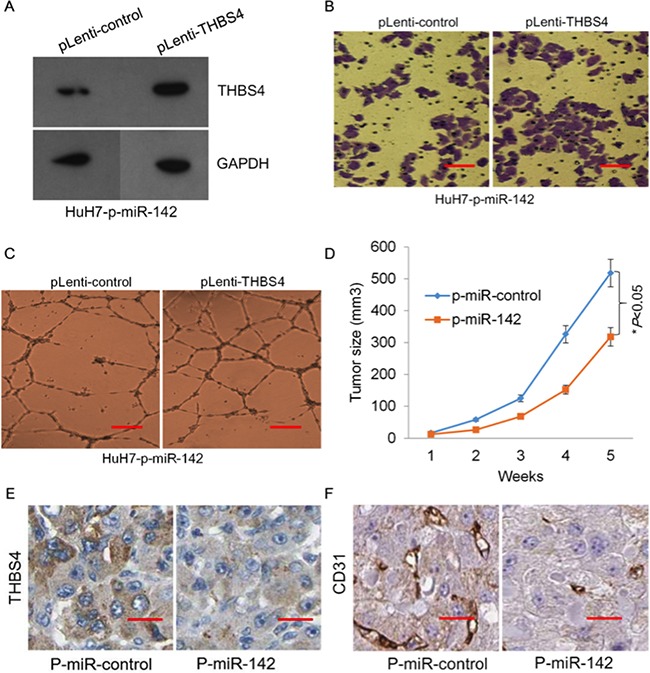
miR-142 inhibits tumor formation of HCC cells through targeting *THBS4* **A**. Western blot of re-expression of THBS4 in stable transfected HuH7-p-miR-142 cells. **B**. Representative images of transwell *in vitro* Matrigel cell invasion in HuH7-p-miR-142 cells expressing THBS4. **C**. Representative tube formation by endothelial cells after incubation with conditioned media (CM) from HuH7-p-miR-142 cells transfected with pLenti-control or pLenti-THBS4 using the tube formation assay. **D**. Tumor growth of xenografts of HuH7-p-miR-control and HuH7-p- miR-142. **E** and **F**. IHC staining of THBS4 and the angiogenesis marker CD31 in tumour tissues from HuH7-p-miR-control and HuH7-p-miR-142 tumor tissues.

To evaluate whether miR-124-dependent inhibition of HCC tumorigenesis was mediated through suppression of *THBS4* expression, we assessed the ability of induced expression of *THBS4* to reverse *in vitro* negative regulation of tumorigenesis by overexpression of miR-142. We ectopically expressed p-miR-142 in HuH7 cells to up-regulate the expression of miR-142, thereby suppressing THBS4 abundance. To these cells, we ectopically expressed control (p-Lenti-control) or THBS4 (p-Lenti-THBS4) into these HuH7-p-miR-142 cells to induce THBS4 expression. We confirmed that ectopic expression of THBS4 in these cells led to a restoration of THBS4 compared to (p-Lenti-control) (Figure 6a). Furthermore, cell migration, invasion and endothelial tube formation (angiogenesis) ability of HuH7 cells was restored upon over-expression of THBS4 (Figure 6b and 6c). These data indicate that miR-142 suppresses HCC tumorigenesis *in vitro* through regulation of *THBS4* gene expression.

To further investigate the effect of miR-142 and THBS4 on tumor growth *in vivo*, we carried out xenograft studies by injecting HuH7-p-miR-control or HuH7-p-miR-142 cells into mice. We observed that stable over-expression of miR-142 in the HuH7 cells significantly inhibited tumor growth in the xenograft tumor models (Figure 6d). Moreover, the IHC staining revealed that expression of THBS4 and the angiogenesis marker CD31 in tumor tissues from HuH7-p-miR-control and HuH7-p-miR-142 xenografts also indicated that over-expression of miR-142 in HuH7 cells resulted in down-regulation of THBS4 and CD31 protein abundance in tumor tissues, which would inhibit angiogenesis of tumors (Figure 6e and 6f).

## DISCUSSION

Here, we identified THBS4 as a target of miR-142 and found that in HCC, miR142 is down-regulated which leads to the overexpression of THBS4, which enhances cell migration, invasion and endothelial tube formation. THBS4 has been reported to regulate tumorigenesis in a variety of tumors. For example, Lin et al reported that the expression of THBS4 was associated with tumor size and TNM staging in human stomach cancer [13]. On the other hand, in breast cancer associated extracellular matrix, McCart Reed *et al* has demonstrated that THBS4 expression contributed to the activated stromal response exhibited during tumor progression and this may facilitate invasion of tumor cells [14]. However, there remains little known about the relationship between *THBS4* expression levels and HCC tumorigenesis. In this study, by comparing the expression of *THBS4* in HCC tumor samples with adjacent normal tissues using qRT-PCR, we found that tumor tissue had higher expression of *THBS4* compared with adjacent normal tissue. We went on to show that depletion of THBS4 studies indicated that over-expression of THBS4 plays a critical role in regulating HCC cell invasion, migration and angiogenesis. Furthermore, we demonstrated that over-expression of *THBS4* correlated with shorter overall survival in HCC patients.

MiR-142 has been reported to regulate various cellular functions during normal cellular growth and proliferation as well as during tumorigenesis [17–19]. Recently, miR-142 was demonstrated as a tumor initiation factor in cervical and testicular germ cell tumors by targeting FZD7 [20, 21]. On the contrary, in other types of tumors such as brain tumors, osteosarcoma and non-small-cell lung carcinoma, miR-142 expression is down-regulated [12, 19, 22]. Increasing evidence also suggests that miR-142 has an effect on epigenetic changes and also regulates autophagy during intestinal inflammation [23, 24]. Utilizing the online bioinformatics database TargetScan, we identified miR-142 as a putative regulator of *THBS4* and this result was confirmed by miRGator 3.0 which suggested an inverse correlation of *THBS4* with miR-142. We further demonstrated that in 30 HCC samples, miR-142 and *THBS4* expression where inversely correlated. We confirmed this inverse correlation by ectopic expression of miR-142, which led to a decrease in *THBS4* expression. Our study also indicated that miR-142 is involved in the invasion and angiogenesis of HCC. Thus, we conclude that over-expression of THBS4 correlates to loss of miR-142 and this pathway contributes to migration and vascular invasion of advanced HCC. Importantly, we also found that the over-expression of THBS4 was correlated to poor prognosis of HCC patients.

Accumulating evidence has suggested that the abnormal expression of miRNAs can result in tumor initiation, invasion and migration [25, 26]. Vascular invasion and metastasis is a major hurdle for current HCC treatments. The ability of tumor cells to undergo migration and invasion enables tumor cells to escape from the primary tumor mass and colonize new environments [27]. Reduced expression of miR-142 was found in some types of cancers such as osteosarcoma and renal cancer [11, 12], but this has not observed in HCC. Previous studies have demonstrated that miRNAs were involved in the regulation of HCC invasion and migration, for example, miR-30, miR-135, and miR-1299 [28–30]. In this study we identified that miR-124 suppresses HCC invasion and migration through down-regulation of *THBS4*, and loss of miR-124 leads to over-expression of *THBS4* thereby promoting HCC invasion and migration.

In summary, we demonstrate that *miR-142* is frequently down-regulated in patients with HCC, and therefore miR-142 may serve as a tumor-suppressing miRNA to suppress HCC initiation and progression. Loss of miR-142 leads to the over-expression of THBS4, which may play a critical role in the regulation of HCC invasion and migration. Furthermore, THBS4 expression levels are highly correlated with the survival of HCC patients. Therefore, our study indicated that miR-142 and/or THBS4 may be promising therapeutic targets for the future development of novel HCC treatments.

## MATERIALS AND METHODS

### Ethics statement

All patient samples used had written informed consent. The study of human samples and animals was approved by the Ethics Committee of Affiliated Hospital of Bengbu Medical College, complying with the Declaration of Helsinki.

### Cell lines culture, tissues and animals

Human HCC cell lines HuH7 and Hep3B were purchased from Cell Bank of the Chinese Academy of Sciences (Shanghai, China). The cells were cultured in PRMI-1640 Medium containing 10% fetal bovine serum with streptomycin (100 μg/mL) and penicillin (100 U/mL). Tumor tissues and adjacent normal tissues were collected in Affiliated Hospital of Bengbu Medical College. None of the patients had received chemotherapy before surgical resection. Mice were bought from Slac animal laboratory company (Shanghai, China).

### RNA isolation and quantitative RT-PCR

Total RNA from cell lines and tissues was isolated using Trizol. The EXPRESS One-Step SuperScript qRT-PCR Kit was used for PCR amplification for the quantification of THBS4 and HPRT1. The following primers were used: THBS4 forward: gcagaaacccagagctgaac, and reverse: agagcatggcagttcttcgt; HPRT1 forward: tgacactggcaaaacaatgca, and reverse: ggtccttttcaccagcaagct. HPRT1 was used as an internal control for normalization. The relative expression was measured by the 2^−ΔCT^ method. miRNA expression was measured with Taqman miRNA assays (Applied Biosystems) using probes for miR-142 (assay ID: 000464) and RNU6 (assay ID: 001973), starting with 10 ng of total RNA, on an ABI Prism 7900 HT. Relative expressions of miR-142 were calculated by normalization to U6.

### IHC

Samples were washed in PBS and sectioned by mesenteric margins and fixed in wooden rafts with the mucosa facing down with the aid of pins. Sections were fixed in 4% paraformaldehyde fixative in 0.1 mol/L sodium phosphate buffer, pH 7.3 at 4°C for 24 h. Following fixation, samples were washed in PBS three times for 10 min. Samples were either stored in PBS containing sodium azide (0.1%) at 4°C for preservation or transferred to PBS + 30% sucrose for 24 h at 4°C for 24 h. Sections were transferred to 50/50 PBS + 30% sucrose/Optimum Cutting Temperature (Tissue Tek) was performed and stored overnight. Samples were sectioned in 10 μm slices at -25°C and mounted on slides, stored at room temperature for 1 h and immersed in 10% normal horse serum solution,1.5% Triton in PBS for 45 min at room temperature. Samples were then incubated with primary antibody for 48 h.

### Transfection of siTHBS4, P-miR-142 and pLenti-THBS4

The hep3B and HuH7 cells were seeded in 24-well plates at 50,000 cells/well and transfected with Endoribonuclease-prepared siTHBS4 or siScramble control and p-miR-142 with or without pLenti-THBS4 (CDS without 3′UTR) using Lipofectamine 3000 (Invitrogen, Carlsbad, CA, USA) according to the manufacturer's protocol. Transfected cells were passaged at 48 hours after transfection and selected with 1ug/mL puromycin to generate stable cell lines.

### Wound healing assay

HCC cells were cultured in 6-well plates overnight. Scratches were created by scratching a straight line with a 20 μl tip vertically in the center of the dish. Cells were washed with PBS to remove the detached cells and images were captured of the scratch. The width of the scratches was observed and measured using Image J v1.47 software (National Institutes of Health, Bethesda, MA, USA). Relative scratch width was calculated normalizing to pre-transfection scratch width.

### Transwell assays

HuH7 and Hep3B cells were plated in the upper chambers of Matrigel-coated wells and growth medium supplemented with 10% serum was added to the lower chamber. After culturing for 24 h, upper chamber was removed and cells in the lower chamber were counted.

### Tube formation assay

The formation of capillary-like structures was evaluated in a 48-well plate by Growth Factor Reduced Matrigel (BD Biosciences). 20,000 cells/well were resuspended in serum-free ECM and plated on solidified Matrigel (200 μl/well). After 8 h of incubation, endothelial cell tubes were imaged in randomly chosen fields. Tubular structures were assessed by manual counting the number of branch points in the 4 randomly chosen low-power fields (×40) from each well.

### Endothelial recruitment assay

24-well Boyden chambers were used for endothelial recruitment assays. HuH7 and Hep3B cells transfected with siScramble or siTHBS4 were cultured in the lower compartments. Fresh 600 μl serumfree medium was added prior to the recruitment experiments. Cells were resuspended in 100 μl serum-free medium and seeded in the upper compartments. After incubation at 37°C for 12 h, upper surface was removed and cells on the lower surfaces were fixed and stained with crystal violet and counted under a light microscope.

### Targeting microRNAs of THBS4 gene prediction

The targeting microRNAs of the *THBS4* gene were predicted by computer-aided algorithms using TargetScan and miRGator 3.0. More detailed information can be acquired from online software TargetScan and miRGator 3.0.

### Luciferase reporter assay

The 3′-UTR sequence of *THBS4* was amplified from human genomic DNA and subcloned into the luciferase reporter vector. HuH7 cells (4 × 10^4^) were seeded in 24-well plates and co-transfected with wild-type (wt) or mutant (mut) 3′-UTR vectors and P-miR-142 or P-miR-control using Lipofectamine 2000. After 48 h, HuH7 cells were assessed for luciferase activity using the Dual-Luciferase Reporter Assay System (Promega) according to the manufacturer's protocol. The experiments were performed in triplicate.

### Western blot

Concentrations of total were determined using the BCA Assay Kit (Thermo). Protein samples were separated on 10% SDS-PAGE and transferred to PVDF membranes (Millipore). Membranes were blocked in 5% non-fat milk for 2 hours and then incubated overnight at 4°C with primary antibody THBS4 (Sigma, 1:1000) and GAPDH (Santa Cruz Biotechnology, 1:1000).

### Statistical analysis

Data is represented as the mean ± SD, and the *p* values determined by two-tailed Student's t-test using SPSS. P < 0.05 was considered as statistically significant.

## SUPPLEMENTARY MATERIALS FIGURES AND TABLES


