# Supplementary material for: Over-expression of Thrombospondin 4 correlates with loss of miR-142 and contributes to migration and vascular invasion of advanced hepatocellular carcinoma
Source: Oncotarget. 2017 Feb 3;8(14):23277–88. doi: 10.18632/oncotarget.15054 (PMC5410303; doi:10.18632/oncotarget.15054)
Supplement: Supplementary file 1 [file oncotarget-08-23277-s001.pdf]

Over-expression of Thrombospondin 4 correlates with loss of miR-142 and contributes to migration and vascular invasion of advanced hepatocellular carcinoma

SUPPLEMENTARY FIGURE

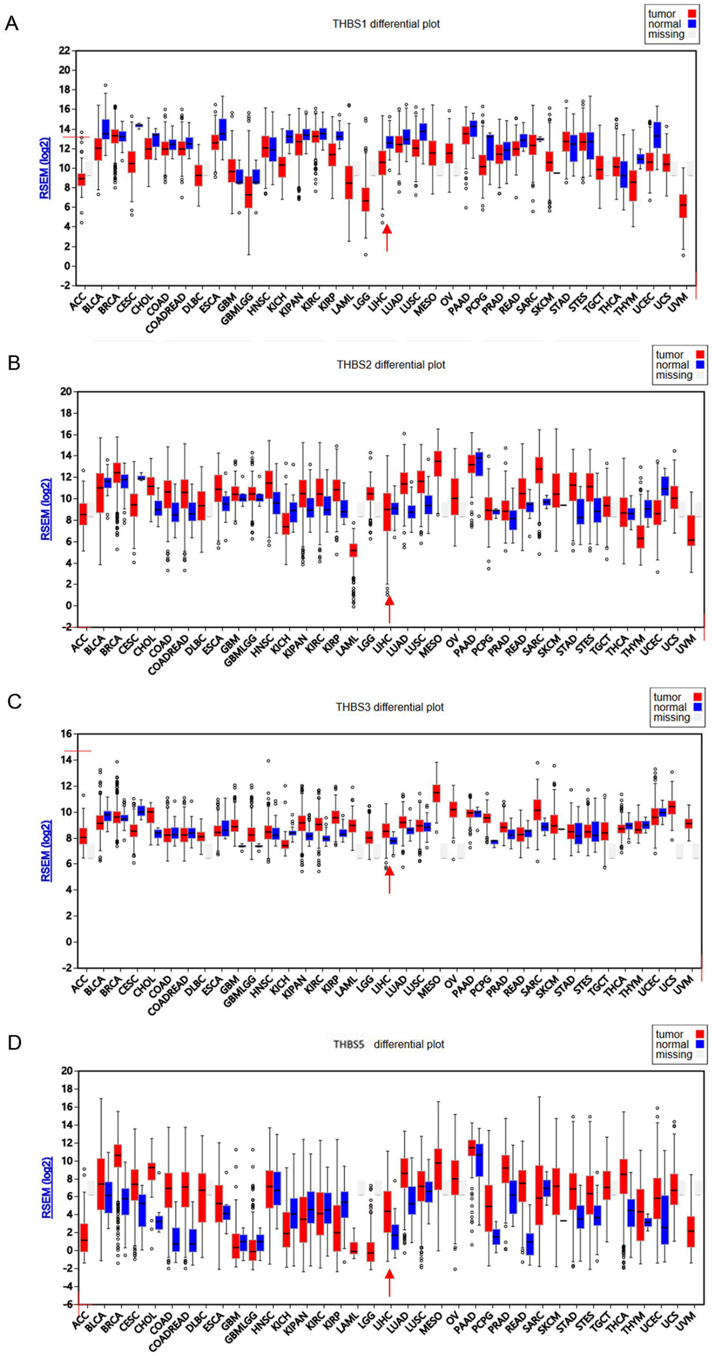

Supplementary Figure 1: Expression of *THBS1, THBS2, THBS3 and THBS5* in HCC of TCGA dataset.
